# Phytochemical composition, hypnotic activity, and antinociceptive properties of cumin essential oil collected from various geographical regions

**DOI:** 10.1002/fsn3.4432

**Published:** 2024-09-18

**Authors:** Abdorahim Nouri, Mansour Mofasseri, Reza Jahani, Masood Ghodrati, Seyed Mohammad Mahdi Emam, Mohammad‐Taghi Ebadi

**Affiliations:** ^1^ Pharmacognosy Department, Faculty of Pharmacy Tehran University of Medical Sciences Tehran Iran; ^2^ School of Chemistry Tehran University Tehran Iran; ^3^ Department of Toxicology and Pharmacology, School of Pharmacy Shahid Beheshti University of Medical Sciences Tehran Iran; ^4^ Department of Horticultural Science, Faculty of Agriculture Tarbiat Modares University Tehran Iran

**Keywords:** cuminaldehyde, *Cuminum cyminum*, essential oil, insomnia, pain

## Abstract

The quantity and quality of the active components of plants are strongly influenced by environmental factors. In this regard, dried cumin seeds were collected from four different locations (SaadatShahr (P1) and Sarvestan (P2) from Fars Province and Kashmar (P3) and Sabzevar (P4) from Khorasan), and their essential oils were isolated by Clevenger apparatus and evaluated using GC and GC–MS. In addition, the hypnotic and antinociceptive activities of the cumin EO sample, which had the highest yield and quality, respectively, were assessed via the pentobarbital‐induced loss of righting test and acetic acid‐induced writhing test. Our results showed that the highest amount of EO was present in sample P4 (3.63%), followed by P3 (2.92%), P2 (2.69%), and P1 (2.31%). GC–MS analysis revealed cuminaldehyde (21.31–33.60%), γ‐terpinene (13.68–23.29%), p‐mentha‐1,4‐dien‐7‐al (14.44–20.84%), p‐mentha‐1,3‐dien‐7‐al (10.06–14.02%), β‐pinene (9.32–11.46%), and p‐cymene (3.16–7.89%) were the major constituents in all the populations. Generally, the results showed that the seeds harvested from areas with hotter and drier climates (P3 and P4) had higher EO yields and cuminaldehyde concentrations but had moderate amounts of γ‐terpinene, β‐pinene, and p‐cymene. In addition, the hypnotic (100 and 200 mg/kg) and antinociceptive (25, 50, and 100 mg/kg) effects of cumin EO were proven in animal models.

## Graphical abstract

The results showed that the environmental factors caused by the cultivation region had a tremendous effect on the quantity and quality of the cumin EO. The cumin seeds harvested from areas with hotter and drier climates had greater EO contents. The EOs of these areas had higher amounts of cuminaldehyde and p‐mentha‐1,4‐dien‐7‐al but had moderate quantities of γ‐terpinene, β‐pinene, and p‐cymene.

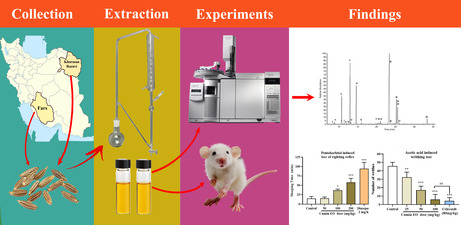

## 1 INTRODUCTION

Since ancient times, the therapeutic value of plant and plant‐derived natural products has led to their wide use in the treatment of different diseases. Because of their enormous chemical composition and diverse spectrum of bioactive constituents, herbs, and spices have played a determinant role in drug research, development, and design (Mathur & Hoskins, 2017). EOs are secondary metabolites that are produced as a plant defense mechanism to respond to stress and prevent herbivores or other animals from relying on them for nourishment. These metabolites are biosynthesized through different parts of plants, including flowers, fruits, peels, seeds, bark, wood, roots, and saps but mostly accumulate in the flowers and leaves of plants. EOs are derived from various chemical sources, including hydrocarbons, oxygenated hydrocarbons, non‐terpenic compounds, isothiocyanates, sulfur, and nitrogenous compounds (Dhifi et al., 2016).

Due to their aroma and wide range of biological activities, EOs are commonly used in the food, cosmetic, perfume, pharmaceutical, and agrochemical manufacturing. In the field of perfumes and cosmetics, EOs are increasingly used to enhance the quality of finished products. In the food industry, EOs are widely used for food preservation (Saeed et al., 2022). EOs have been extensively applied in traditional and folk medicine due to the presence of a myriad of different bioactive phytochemicals and various biological activities, including anti‐inflammatory, immunomodulatory, antibacterial, antiviral, antifungal, antirheumatic, antioxidant, expectorant, sedative, and antitussive properties (Dhifi et al., 2016). Moreover, EOs have been traditionally used for their carminative, anti‐inflammatory, analgesic, antispasmodic, diaphoretic, diuretic, and emmenagogue activities. In addition, EOs have external uses because of their astringent, antiseptic, antipyretic, and antimicrobial properties (Sharifi‐Rad et al., 2018). Extracted EOs from separated parts of plants have demonstrated extensive biological effects, including antirheumatic, antiviral, antibacterial, antioxidant, antispasmodic, antidiabetic, anti‐inflammatory, anti‐Alzheimer's, anticancer, antiobesity, antifungal, and detoxification activities (Baptista‐Silva et al., 2020; Sharifi‐Rad et al., 2018).

The Apiaceae family, which includes approximately 2786 species and 347 genera, is one of the main flowering plant families worldwide. The species of this family include vegetables, culinary plants, and medicinal plants. The Apicaceae family is native to India, Iran, Egypt, and South Asia. In Iran, Apiaceae is represented by 121 genera, which belong to 360 species (Mozaffarian, 2007).

Cumin (*Cuminum cyminum* L.) is one of the oldest and most commonly used aromatic species for which dried seeds are applied as spices. Cumin is an annual glabrous plant up to 60 cm in height. In Iran, it is generally called cumin or zirech‐e sabz and is known as cumin, zeera, comino, zira, kummel, kemon, or kamun in other regions of the world. The surface of the plant contains five primary ridges replaced with four secondary ridges bearing numerous small hairs. The blue‐green linear leaves are glabrous and 5–10 cm in length. The leaves contained oblong linear tips, of which the lower tips were mostly doubly trifoliate. The glabrous slender stem is separated into two similar parts. Each cumin branch consisted of 3–9 umbels with 5–7 umbellets. The white or pink flowers bloom in small compound umbels and branch out in groups of 3–5. The fruit is a lateral schizocarp approximately 4–6 mm long and 1.5 mm wide and contains a single seed. The seeds are 6 mm long and oblong, but they are thicker in the middle. The seeds commonly germinate at low temperatures (below 20°C), and their germination is stopped at higher temperatures. Some seeds have a short stalk (Piri et al., 2019; Soltani et al., 2019). The channels around the seed (oil ducts = vitta) are the places where the EO accumulates in cumin fruits (Tabarsa et al., 2020).

In Iran, cumin is traditionally used for its carminative, antispasmodic, astringent, analgesic, stimulant, and antidiabetic effects. It is also applied for the treatment of digestive disorders and coughs (Al‐Snafi, 2016). This plant is used as a medicine for curing dyspepsia, toothache, indigestion, hoarseness, stomach pain, hypertension, scorpion bites, weight loss, and jaundice in folk medicine (Taghizadeh et al., 2016). Cumin fruits are used as a medicine for colic, diarrhea, dyspepsia, and flatulence, as well as for stimulating breast milk production in Iranian traditional medicine (Belal et al., 2017).

Phytochemical analysis of different reports has demonstrated that cumin is a rich source of different phytoconstituents, including alkaloids, anthraquinones, coumarins, glycosides, flavonoids, proteins, saponins, resins, tannins, dietary fibers, steroids, minerals, fats (especially monounsaturated fat), vitamin A, vitamin B (thiamin, niacin, vitamin B6, riboflavin), vitamin C, and vitamin E (Hussain et al., 2008).

**FIGURE 1:**
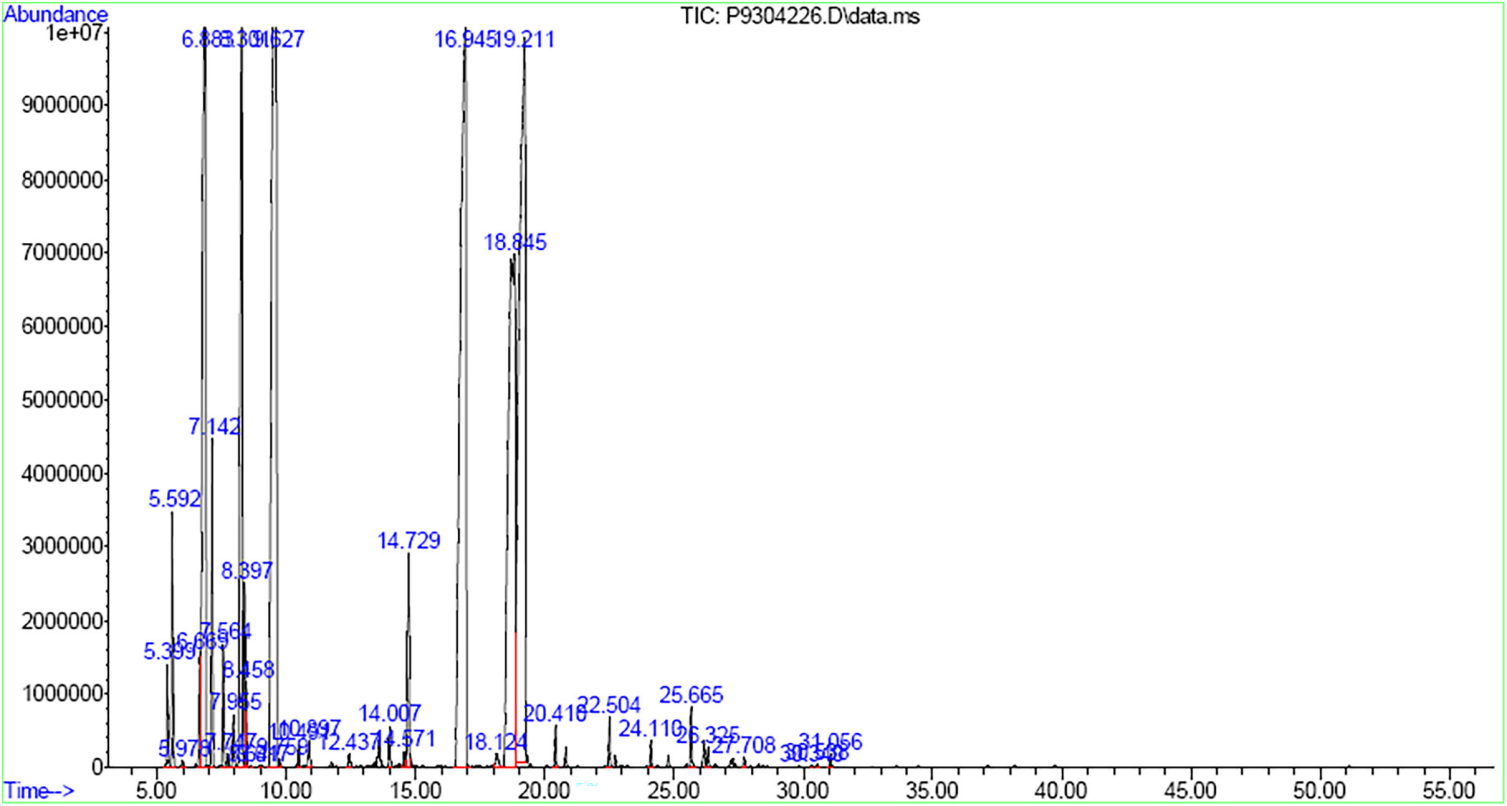
GC–MS chromatogram of cumin EO.

Many genetic and environmental factors, namely, climate, geographic conditions, seasonal variations, and growth stage, may extensively affect the chemical composition and, consequently, the biological activities and commercial use of medicinal and aromatic plants (Li & Jiang, 2004). As described by Moghaddam and Pirbalouti (2017), morphological and phytochemical varieties of 20 genotypes of Iranian cumin and 23 constituents were recognized in the EOs, and γ‐terpinene, cuminaldehyde, cumin alcohol, and β‐pinene were the main constituents of all the genotypes. EOs extracted from the seeds of three native cumin varieties from Morocco were analyzed, and the major constituents of all the considered EOs were cuminaldehyde, γ‐terpinen‐7‐al, α‐terpinen‐7‐al, γ‐terpinene, β‐cymene, β‐pinene, and p‐mentha‐1,4‐dien‐7‐ol (Hajlaoui et al., 2010). As described by Hajlaoui et al. (Li & Jiang, 2004), the phytochemical analysis and biological activities of Tunisian cumin EO from seeds and 21 constituents were identified, and cuminaldehyde, γ‐terpinene, o‐cymene, *β*‐pinene, 2‐caren‐10‐al, *trans*‐carveol, and myrtenal were the main constituents. The EO of the cumin seeds from China was investigated GC and GC–MS; 37 constituents were measured, of which cuminaldehyde, cuminic alcohol, *γ*‐terpinene, safranal, p‐cymene, and β‐pinene were the main constituents (Rana, 2014). In another study, the EO composition of cumin seeds was determined. Twenty‐six components were identified as cuminaldehyde, p‐cymene, β‐pinene, α‐terpinen‐7‐al, γ‐terpinene, p‐cymen‐7‐ol, and thymol (Petretto et al., 2018). The main compounds of EO found in the Turkish cumin seed were cuminaldehyde, p‐mentha‐l,4‐dien‐7‐al, p‐mentha‐l,3‐dien‐7‐al, p‐cymene, α‐terpinene, and β‐pinene (Naeini et al., 2014). GC–MS analysis of cumin EOs revealed the identification of 17 components, the main of which were α‐pinene, limonene, and 1,8‐cineole (Kedia et al., 2014). The analysis of cumin EO shows that cymene, cuminaldehyde, γ‐terpinene, and β‐pinene were the main components (Cartwright, 2016; Figure 1).

Iran is the second largest producer of cumin after India and accounts for approximately 4% of the global production (30,000 tons; www.trademap.org). Considering the widespread cultivation of cumin in Iran (due to the capability of rainfed cultivation and low water consumption) and the high production of this product in some provinces, a comparison of the quality of the produced plant material according to international standards such as ISO 9301 is necessary. Therefore, this investigation was conducted to examine the quality of cumin seeds produced in different regions of the Khorasan Razavi and Fars provinces of Iran. In addition, the cumin seed sample, which had the highest EO content and quality, was chosen for pharmacological activity evaluation in animal models. In this regard, the hypnotic and antinociceptive effects of the cumin seed EO were assessed via the pentobarbital‐induced loss of righting test and the acetic acid‐induced writhing test, respectively.

## 2 MATERIALS AND METHODS

### 2.1 Chemicals and reagents

Sodium pentobarbital, diazepam, acetic acid, and polyoxyethylene‐sorbitan monolated (Tween 80) were obtained from Sigma–Aldrich Chemical Co. (St. Louis, Missouri, USA). Pentobarbital and diazepam were dissolved in normal saline, while the cumin seed EO was emulsified in Tween 80 and water for pharmacological studies. All the solvents were of analytical grade and were obtained from Merck Co. (Darmstadt, Germany).

### 2.2 Plant materials

**FIGURE 2:**
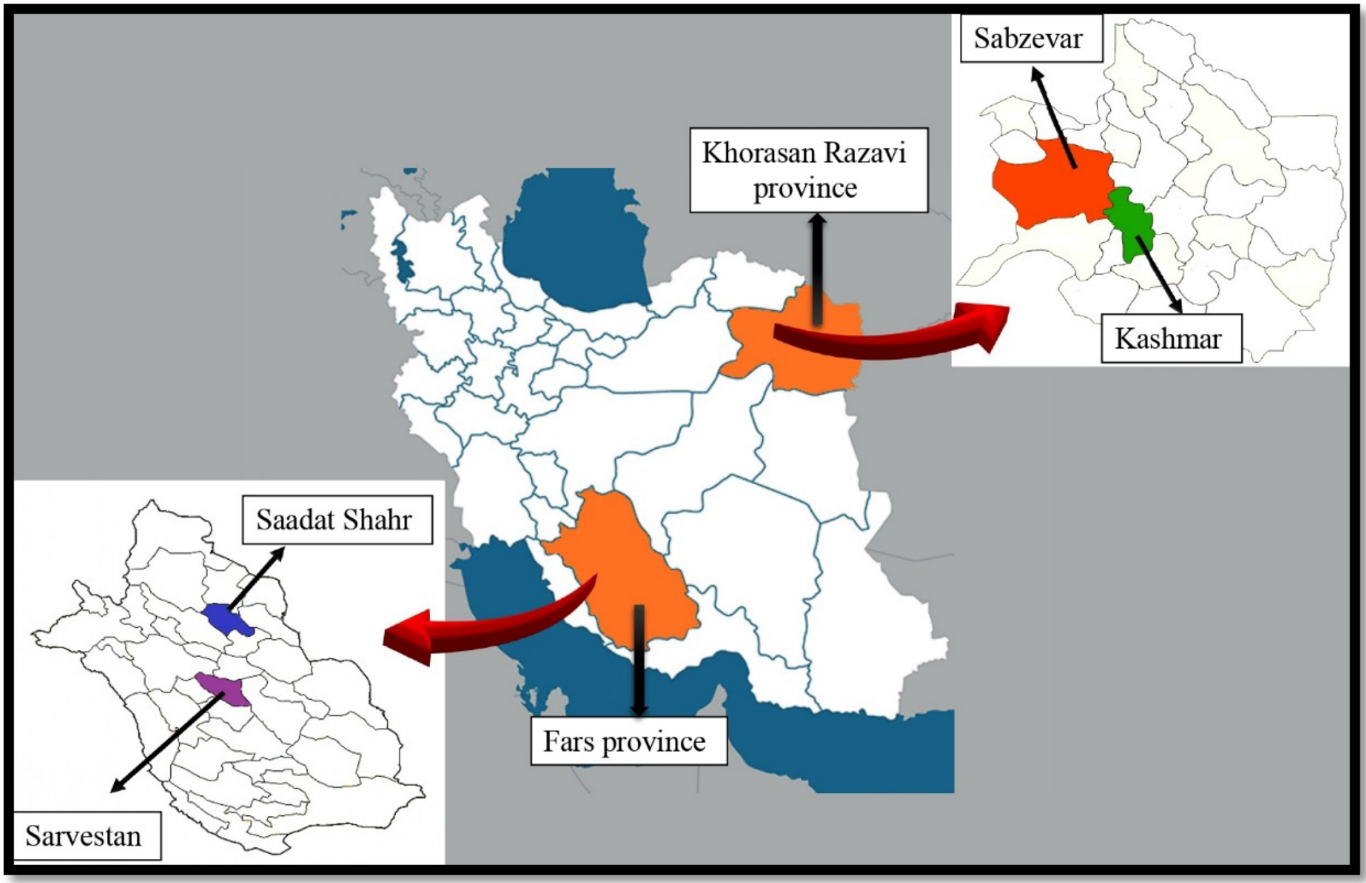
Different sampling locations of cumin from Khorasan Razavi and Fars provinces.

*Cuminum cyminum* L. seeds were collected from four different geographical regions of Iran, namely, Saadat Shahr (P1) and Sarvestan (P2) in Fars Province (southern Iran) and Kashmar (P3) and Sabzevar (P4) in Khorasan Razavi Province (northeastern Iran), during September 2022 (Figure 2). All specimens were authenticated at the Herbarium of the Faculty of Pharmacy, Tehran University of Medical Sciences, Tehran, Iran. The voucher specimens of the plants were then deposited in the herbarium of the Faculty of Pharmacy, Department of Pharmacognosy, Tehran University of Medical Sciences (Tehran, Iran). The geographical properties of the cumin planting areas are illustrated in Table 1.

**TABLE 1:** Geographical properties of cumin planting areas.

Plant sample	Herbarium code	The average annual rainfall (mm)	The mean annual temperature (°C)	Latitude (E)	Longitude (N)	Altitude (m)
P_1_	PMP‐3674	368.2	17.2	53°1400′	30°0799′	1796
P_2_	PMP‐3675	346.8	18.45	53°2204′	29°2737′	1557
P_3_	PMP‐3672	189.4	17.82	58°6458′	35°3687′	1063
P_4_	PMP‐3673	177.2	16.75	57°6667′	36°2167′	978

*Note*: P_1_ (Saadat Shahr); P_2_ (Sarvestan); P_3_ (Kashmar); P_4_ (Sabzevar).

### 2.3 EO extraction

The seeds of each sample were crushed separately and subjected to hydro‐distillation for 3 h using a Clevenger‐type apparatus (Adams, 2017). The EOs were dehydrated over anhydrous sodium sulfate and preserved at 4 ***°*** C until examination.

### 2.4 Gas chromatography and gas chromatography–mass spectrometry analysis

The EOs composition analysis was done by GC and GC/MS using the same equipment and method described by Moradi‐Sadr et al. (2023). Identification of the constituents was carried out via computer matching with the Wiley libraries, as well as by comparison of Kovats indices (KI) and patterns of mass fragmentation with those published for standard compounds. The reported KI of each identified compound for the HP‐5 column was found via the identification of EO constituents via a gas chromatography–mass spectrometry book and the National Institute of Standards and Technology (NIST) website. The KI (based on retention indices, KIc) was calculated based on the following formula (Rt: Retention time):

$$\begin{array}{c} \mathrm{KIc}=100\\ \times \left[n+\left(\mathrm{Rt}\;\text{compound}-\mathrm{Rt}\;\text{smaller alkane}/\mathrm{Rt}\;\text{large alkane}-\mathrm{Rt}\;\text{smaller alkane}\right)\right] \end{array}$$

### 2.5 Animals and treatments

Male albino NMRI mice weighing >20–25 g were kept in a regular laboratory environment (12 h light/dark cycle, 22 ± 2°C temperature, and 10% humidity). Mice were fed a normal laboratory rodent diet ad libitum. All treatments were administered intraperitoneally at an injection volume of 10 mL/kg by European Community guidelines. In addition, all protocols were approved by the Animal Experimentation Committee.

### 2.6 Evaluation of hypnotic activity

The pentobarbital‐induced loss of the righting reflex test was conducted as previously described for the assessment of the hypnotic effects of the cumin seed EO. The mice were divided into five groups (*n* = 10). Group 1, the negative control group, received normal saline, while groups 2–4 received different doses of EO (50, 100, and 200 mg/kg). Group 5 was subjected to diazepam (2 mg/kg) as the positive control group. After 30 min, all the groups were treated with sodium pentobarbital (45 mg/kg), and the duration of sleep (the period between the loss and recovery of the righting reflex) was recorded. All the experiments were performed between 9 a.m. and 4 p.m. (Jahani et al., 2022).

### 2.7 Assessment of antinociceptive activity

An acetic acid‐induced writhing test was performed to evaluate the antinociceptive activity of the strains. Mice were randomly divided into five groups (*n* = 10). Group 1, the negative control group, received normal saline, whereas groups 2–4 were administered different doses of cumin seed EO (25, 50, or 100 mg/kg). Group 5 was subjected to celecoxib (80 mg/kg) as the reference drug. After 30 min, all the groups were treated with 0.1 mL of acetic acid (1% v/v) as an irritant stimulant, and after a latency period of 5 min, the number of writhes (abdominal constriction and stretching of at least one hind limb) was counted for 25 min. A reduction in the total number of observed writhes was considered antinociceptive activity (Jahani et al., 2022).

### 2.8 Statistical analysis

The data were analyzed using GraphPad Prism software version 8.0 for Windows (San Diego, CA, USA). The results obtained from behavioral tests are expressed as the mean ± standard error of the mean (SEM). Comparisons between groups were made by one‐way analysis of variance (ANOVA) followed by Tukey's multiple comparison test when the data included three or more groups; *p‐*values less than .05 were considered to indicate significant differences.

## 3 RESULTS

### 3.1 EO content

As shown in Table 2, the EO content ranged from 2.31% to 3.63% (v/w) as a function of the area of interest. P4 had the highest EO content (3.63%), followed by P3 (2.92%) and P2 (2.69%), while the lowest content of EO was observed in P1 (2.31%).

**TABLE 2:** EO content (%) of cumin seed samples.

Plant sample	EO content
P_1_	2.28 ± 0.07 a
P_2_	2.66 ± 0.06 b
P_3_	2.90 ± 0.09 c
P_4_	3.64 ± 0.11 d

*Note*: Results are provided as mean ± SD; P_1_ (Saadat Shahr); P_2_ (Sarvestan); P_3_ (Kashmar); P_4_ (Sabzevar); Means followed by a common letter are not significantly different.

### 3.2 EO composition

**FIGURE 3:**
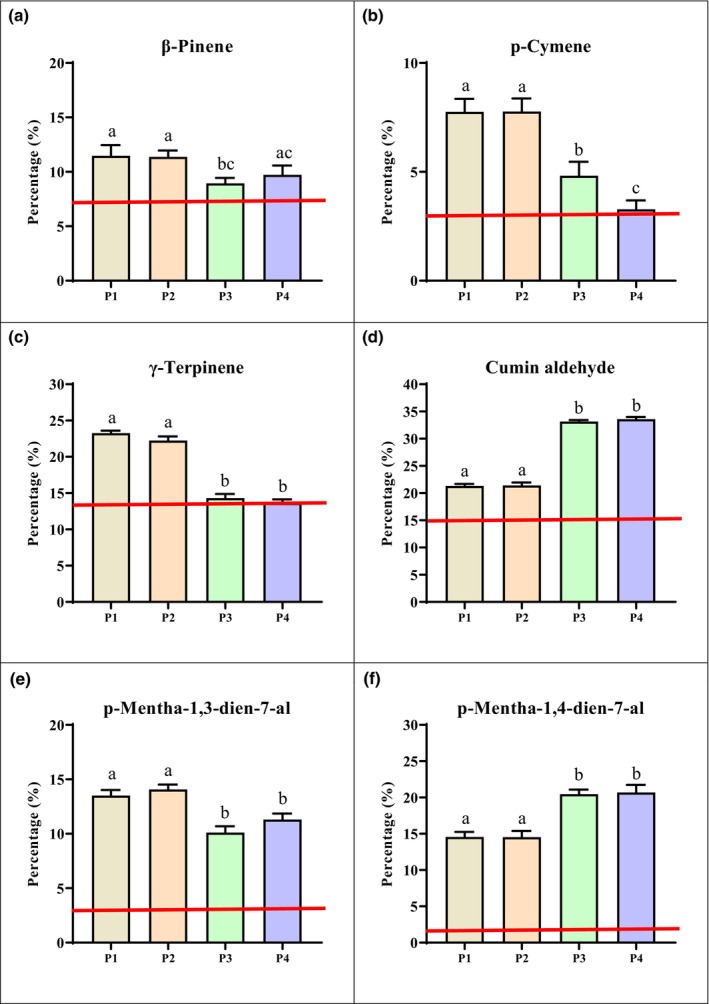
Percentage of β‐pinene (a), p‐cymene (b), γ‐terpinene (c), Cuminaldehyde (d), p‐mentha‐1,3‐dien‐7‐al (e), and p‐mentha‐1,4‐dien‐7‐al (f) at different planting regions. P1 (Saadat Shahr); P2 (Sarvestan); P3 (Kashmar); P4 (Sabzevar); Results are provided as mean ± SD; Means followed by a common letter are not significantly different. The red line in each diagram represents the minimum amount of the desired compound according to the ISO 9301.

The results of GC–MS analysis of cumin seed EOs extracted from four different populations (P1, P2, P3, and P4) are shown in Table 3 and Figure 3. According to the results, the major compounds were cuminaldehyde (21.31–33.60%), γ‐terpinene (13.68–23.29%), p‐mentha‐1,4‐dien‐7‐al (14.44–20.84%), p‐mentha‐1,3‐dien‐7‐al (10.06–14.02%), β‐pinene (9.32–11.46%), and p‐cymene (3.16–7.89%) in all populations. In the population of Saadat Shahr (P1), the main compounds were γ‐terpinene (23.29%), cuminaldehyde (21.31%), p‐mentha‐1,4‐dien‐7‐al (14.71%), p‐mentha‐1,3‐dien‐7‐al (13.58%), β‐pinene (11.46%), and p‐cymene (7.64%), which composed 98.18% of the EOs in this population. γ‐Terpinene (22.11%), cuminaldehyde (21.47%), p‐mentha‐1,4‐dien‐7‐al (14.44%), p‐mentha‐1,3‐dien‐7‐al (14.02%), β‐pinene (11.23%), and p‐cymene (7.89%) were the major compounds in the EOs from the Sarvestan (P2) sample, and these phytochemicals contained 98.21% of the EOs. The analysis of the chemical composition of EOs from Kashmar (P3) showed that cuminaldehyde (33.10%) was the most abundant compound among the identified compounds in this population. p‐mentha‐1,4‐dien‐7‐al (20.46%), γ‐terpinene (14.13%), p‐mentha‐1,3‐dien‐7‐al (10.06%), β‐pinene (9.32%), and p‐cymene (4.95%) were the other main constituents in the chemical composition of EOs from Kashmar (P3). The mentioned compounds composed 98.41% of the EOs extracted from seeds from P3. As a result of phytochemical assessment of the EOs extracted from the Sabzevar population (P4), cuminaldehyde (33.60%), p‐mentha‐1,4‐dien‐7‐al (20.84%), γ‐terpinene (13.68%), p‐mentha‐1,3‐dien‐7‐al (11.19%), β‐pinene (9.70%), and p‐cymene (3.16%) were recognized as the greatest abundant compounds, accounting for 90.55% of the whole EOs obtained from this population. The percentage of identified compounds also agreed with the chromatographic profile of the International Standards Organization (ISO: 9301/2003) for the EOs of cumin seeds (Table 4).

**TABLE 3:** EOs composition (%) of cumin seed samples in comparison with ISO standard (9301/2003).

No.	Compounds	KI	RI	RTx	P_1_	P_2_	P_3_	P_4_	ISO standard
1	α‐thujene	923	924	5.4	0.42 ± 0.02	0.53 ± 0.03	0.38 ± 0.01	0.36 ± 0.02	–
2	α‐pinene	930	931	5.6	0.93 ± 0.03	1.39 ± 0.03	0.88 ± 0.02	0.86 ± 0.02	–
3	Sabinene	969	971	6.6	0.91 ± 0.02	0.71 ± 0.05	0.94 ± 0.05	0.94 ± 0.06	–
4	β‐pinene	974	976	6.7	11.47 ± 0.98	11.38 ± 0.58	8.94 ± 0.50	9.73 ± 0.85	7.0–20.0
5	Myrcene	986	988	7.1	0.71 ± 0.05	0.97 ± 0.04	0.67 ± 0.05	0.69 ± 0.06	–
6	α‐phellandrene	1003	1004	7.5	1.33 ± 0.04	0.64 ± 0.06	1.36 ± 0.07	1.29 ± 0.09	–
7	α‐terpinene	1013	1015	7.9	0.39 ± 0.04	0.34 ± 0.05	0.34 ± 0.03	0.34 ± 0.04	–
8	p‐cymene	1022	1023	8.2	7.75 ± 0.60	7.77 ± 60	4.82 ± 0.64	3.28 ± 0.41	3.0–17.0
9	Limonene	1025	1026	8.3	0.38 ± 0.06	0.49 ± 0.06	0.18 ± 0.05	0.14 ± 0.03	–
10	β‐phellandrene	1026	1027	8.4	0.2 ± 0.03	0.21 ± 0.03	0.45 ± 0.04	0.32 ± 0.04	–
11	1,8‐cineole	1028	1029	8.4	0.18 ± 0.05	0.29 ± 0.05	0.19 ± 0.05	0.21 ± 0.03	–
12	γ‐terpinene	1056	1058	9.5	23.27 ± 0.35	22.24 ± 0.57	14.31 ± 0.57	13.63 ± 0.50	14.0–32.0
13	Terpinene‐4‐ol	1172	1174	14.0	0.17 ± 0.04	0.25 ± 0.03	0.16 ± 0.03	0.21 ± 0.04	–
14	p‐menth‐3‐en‐7‐al	1189	1191	15.0	0.24 ± 0.04	0.75 ± 0.06	0.46 ± 0.05	0.74 ± 0.05	–
15	Cuminaldehyde	1238	1239	16.7	21.31 ± 0.37	21.41 ± 0.53	33.15 ± 0.27	33.57 ± 0.4	15.0–46.0
16	p‐mentha‐1,3‐dien‐7‐al	1279	1282	18.5	13.51 ± 0.51	14.07 ± 0.45	10.11 ± 0.58	11.3 ± 0.56	2.8–22.0
17	p‐mentha‐1,4‐dien‐7‐al	1289	1291	18.9	14.57 ± 0.70	14.53 ± 0.87	20.47 ± 0.63	20.68 ± 1.05	1.5–16.0
18	(E)‐β‐farnesene	1451	1454	25.6	0.28 ± 0.06	0.37 ± 0.05	0.34 ± 0.04	0.26 ± 0.05	–
	Total identified	–	–	–	98.02	98.34	98.15	98.55	–

*Note*: Results are provided as mean ± SD. P_1_ (Saadat Shahr); P_2_ (Sarvestan); P_3_ (Kashmar); P_4_ (Sabzevar).

Abbreviations: KI, Kovats index, RI, Retention index.

**TABLE 4:** A summary of the characteristics of cumin EO components (%) in ISO standard (9301/2003).

Component	Minimum	Maximum
β‐pinene	7.0	20.0
γ‐terpinene	14.0	32.0
*p*‐cymene	3.0	17.0
Cuminic aldehyde	15.0	46.0
*p*‐mentha‐1,3‐dien‐7‐al	2.8	22.0
*p*‐mentha‐1,4‐dien‐7‐al	1.5	16.0

**FIGURE 4:**
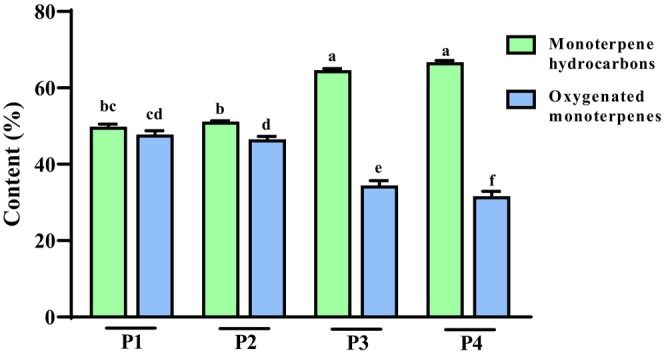
Evaluation of EOs chemical groups in planting regions. Results are provided as mean ± SD; P1 (Saadat Shahr); P2 (Sarvestan); P3 (Kashmar); P4 (Sabzevar); Means followed by a common letter are not significantly different.

Cuminaldehyde (33.10–33.60%) was the most common compound in the analyzed EOs of the P3 and P4 samples. Accordingly, the highest concentrations of this compound were detected in P4 (33.60%) and P3 (33.10%), while P2 (21.47%) and P1 (21.31%) had the highest concentrations. γ‐Terpinene was the most abundant compound in the P1 (23.29%) and P2 (22.11%) populations. The results confirmed the effect of the environment and climate on the EO content. The mean annual temperature and average annual rainfall in these two populations were significantly lower than those in the P1 and P2 samples, and these two factors markedly contributed to the higher quality of EOs in P3 and P4. These results are those reported in the literature, which confirmed that various samples from different localities contain varied EOs and phytochemicals (Koohsari et al., 2020; Li & Jiang, 2004; Mathur & Hoskins, 2017; Moghaddam & Pirbalouti, 2017). Monoterpenes and oxygenated monoterpenes were the main constituents in the cumin EO (Figure 4).

### 3.3 Hypnotic activity of cumin EO

**FIGURE 5:**
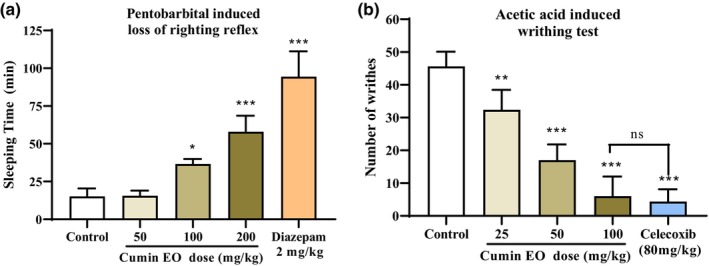
Hypnotic (a) and antinociceptive (b) activities of cumin EO (Essential oil; F4) in the pentobarbital‐induced loss of righting reflex test and acetic acid‐induced writhing test, respectively. Values are expressed as mean ± SEM. *p‐value <.05, **p<.01, and ***p‐value <.001 indicate significant differences compared to the control group. ns refers to no significant difference level between the indicated groups. (*n* = 10) in each group.

Due to the beneficial effects of plants in the treatment of various diseases, as well as their fewer side effects than synthetic drugs, the herbal medicine market is growing at a significant rate. Since plants containing more EOs are more cost‐effective at producing herbal medicinal products, sample P4 was chosen for investigating the pharmacological effects of the cumin EO. The results of the hypnotic activity of the cumin EO are illustrated in Figure 5. A. Treatment of mice with 100 and 200 mg/kg cumin EO significantly increased sleep duration (36.6 ± 1.5 and 58.0 ± 4.7 min, respectively) compared to that of the negative control group (15.2 ± 2.3 min), for which the *p*‐values were less than .05 and .001, respectively. However, cumin EO was not effective at 50 mg/kg (*p*‐values >.999). The sleep duration of diazepam‐treated mice (2 mg/kg), which served as a reference control, was significantly greater (94.38 ± 5.9 min) than that of the negative control group (*p‐*values <.001).

### 3.4 Antinociceptive activity of cumin EO

The antinociceptive effects of different doses of cumin EO on the writhing test are illustrated in Figure 3b. Cumin EO at 25, 50, and 100 mg/kg significantly decreased the writhing response induced by the administration of acetic acid solution compared to that in the negative control group as follows: 25 mg/kg (28.94% reduction, *p‐*value <.01), 50 mg/kg (62.72% reduction, *p‐*value <.001), and 100 mg/kg (86.84% reduction, *p‐*value <.001). In addition, compared with the negative control, celecoxib (80 mg/kg), a nonsteroidal anti‐inflammatory drug, significantly reduced the writhing response. However, there was no difference between the effects of 100 mg/kg cumin EO and 80 mg/kg celecoxib.

## 4 DISCUSSION

Many internal and external features, including genotype, geographical location, climatic situations, growing time, and the techniques used for EO extraction, affect the EO content of medicinal plants (Dubey et al., 2017). Previous studies have shown that the cumin EO content is influenced by factors such as the cultivation region, harvesting time, extraction technique, and storing conditions (15). Our results showed that the seeds harvested from areas with hotter and drier climates (P3 and P4) had greater EO contents. A direct relationship between the increase in environmental stresses and the quantity and quality of active substances (especially EOs) in medicinal plants has been observed since these compounds are synthesized as protective agents against stresses in plants and have defensive and life‐preserving functions (Zargoosh et al., 2021).

The results are those reported in the literature, which confirmed that various samples from different localities contain varied EOs and phytochemicals (Koohsari et al., 2020; Li & Jiang, 2004; Mathur & Hoskins, 2017). Monoterpenes hydrocarbons and oxygenated monoterpenes were the main constituents in the cumin EO (Figure 4). In other investigations, monoterpenes were also stated as the most abundant chemical constituents in cumin EO (Jahani et al., 2022).

This study showed that key compounds of cumin EO such as β‐pinene, p‐cymene, γ‐terpinene, and cuminaldehyde are particularly sensitive to environmental factors. Cuminaldehyde was the most abundant compound in the P3 and P4 populations. The cuminaldehyde possesses a nice aroma and adds to the special smell of cumin. Cuminaldehyde material is commercially available for the fragrance and cosmetics industries. Cumin seeds with high cuminaldehyde contents are more desirable than seeds with high other monoterpenes content in different markets (Dubey et al., 2017). Some studies indicated that higher temperatures can increase the concentration of cuminaldehyde in cumin seeds. For example, a research conducted in arid regions demonstrated that increased sunlight and reduced water availability led to a higher synthesis of cuminaldehyde (Moghaddam & Pirbalouti, 2017). Conversely, cooler and wetter climates tend to reduce the concentration of this compound (Hazrati et al., 2024). Many investigations have reported a progressive and significant correlation between EO percentage and cuminaldehyde amount in cumin (Abbdellaoui et al., 2019; Dubey et al., 2017). Therefore, according to their EO constituents, the cumin varieties were clustered into two chemotypes, high cuminaldehyde and low cuminaldehyde (with high amounts of other monoterpenes), indicating a significant variance in EO quality.

β‐pinene, another key component of cumin EO, is known for its antimicrobial properties. Similar reports with cuminaldehyde have been published about this compound. A study reported that cumin plants grown under higher temperature regimes showed an increased synthesis of β‐pinene (Li et al., 2009). However, excessive heat stress could lead to a decrease in overall oil yield, despite the higher β‐pinene content (Naeini et al., 2014). p‐Cymene, which contributes to the characteristic spicy aroma of cumin, is also influenced by climatic conditions. Optimal levels of p‐cymene are often achieved under moderate temperatures and adequate water supply (Fakhari et al., 2005). A research has shown that cumin plants under moderate water stress conditions produced essential oil with higher p‐cymene concentration, suggesting that controlled water stress can be beneficial for this compound's synthesis (Jhalegar et al., 2014). γ‐Terpinene, known for its antioxidant properties, is sensitive to both temperature and water availability. Studies have demonstrated that warmer temperatures and moderate water stress can enhance γ‐terpinene levels in cumin essential oil. For instance, cumin grown in regions with high daytime temperatures and limited irrigation showed an increase in γ‐terpinene content, likely due to the plant's adaptive response to stress (Karydogianni et al., 2022). However, extreme drought conditions could lead to a decrease in both the yield and quality of the essential oil, including its γ‐terpinene content (Rasouli et al., 2023).

Most herbal remedies for the treatment of insomnia reduce sleep latency and increase sleep duration through interactions with different brain neurotransmitter systems, such as gamma‐aminobutyric acid (GABA). This is not surprising since different subtypes of GABA receptors are recognized as the main receptors responsible for the treatment of insomnia and seizures (Bruni et al., 2021). Although there are no reports on the hypnotic effects of cumin EO, its anticonvulsant activity has been proven in previous studies (Janahmadi et al., 2006). Considering that GABA receptors play a significant role in the control of epilepsy, the hypnotic effect of the cumin EO observed in the present study could be at least in part attributed to the interaction of the main EO compounds, such as cuminaldehyde, with GABA receptors.

As described by Koohsari et al. (2020), the antinociceptive activity of cuminaldehyde was reported as the main component of cumin seeds. In their study, intraperitoneal administration of cuminaldehyde at 25 and 50 mg/kg significantly decreased the number of writhes. The findings obtained with 50 mg/kg cuminaldehyde in their study were similar to the results obtained with 100 mg/kg cumin EO in our study. Although cuminaldehyde constitutes 33% of cumin EO, the findings of the current investigation are in line with the results achieved by Koohsari et al. considering that a set of compounds in the EO is responsible for the observed effect.

## 5 CONCLUSION

The results showed that the environmental factors caused by the cultivation region had a tremendous effect on the quantity and quality of the cumin EO. The cumin seeds harvested from areas with hotter and drier climates (P3 and P4) had greater EO contents. The EOs of these areas had higher amounts of cuminaldehyde and p‐mentha‐1,4‐dien‐7‐al but had moderate quantities of γ‐terpinene, β‐pinene, and p‐cymene. In addition, cumin EO has shown significant hypnotic and antinociceptive effects in animal models. Therefore, these places are recommended for the development of cumin cultivation in Iran for commercial use and therapeutic purposes.

## AUTHOR CONTRIBUTIONS

**Abdorahim Nouri:** Conceptualization (equal); data curation (equal); formal analysis (equal); investigation (equal); methodology (equal); software (equal); writing – original draft (equal). **Mansour Mofasseri:** Data curation (equal); formal analysis (equal); software (equal); writing – original draft (equal). **Reza Jahani:** Data curation (equal); formal analysis (equal); investigation (equal); software (equal); writing – original draft (equal). **Masood Ghodrati:** Software (equal); visualization (equal). **Seyed Mohammad Mahdi Emam:** Data curation (equal); formal analysis (equal); investigation (equal); software (equal); writing – original draft (equal). **Mohammad‐Taghi Ebadi:** Conceptualization (equal); funding acquisition (equal); investigation (equal); methodology (equal); project administration (equal); resources (equal); supervision (equal); writing – review and editing (equal).

## CONFLICT OF INTEREST STATEMENT

The authors declare no conflict of interest.

## Data Availability

The data that support the findings of this study are available on request from the corresponding author.
